# Spatial metabolomics in mental disorders and traditional Chinese medicine: a review

**DOI:** 10.3389/fphar.2025.1449639

**Published:** 2025-01-31

**Authors:** Chaofang Lei, Jiaxu Chen, Zhigang Chen, Chongyang Ma, Xudong Chen, Xiongxing Sun, Xukun Tang, Jun Deng, Shiliang Wang, Junlin Jiang, Dahua Wu, Le Xie

**Affiliations:** ^1^ Department of Neurology, Hunan Provincial Hospital of Integrated Traditional Chinese and Western Medicine (The Affiliated Hospital of Hunan Academy of Traditional Chinese Medicine), Changsha, China; ^2^ School of Traditional Chinese Medicine, Beijing University of Chinese Medicine, Beijing, China; ^3^ Department of Neurology, Dongfang Hospital, Beijing University of Chinese Medicine, Beijing, China; ^4^ School of Traditional Chinese Medicine, Capital Medical University, Beijing, China; ^5^ Department of Psychiatry, The Second Xiangya Hospital of Central South University, Changsha, China

**Keywords:** mental disorders, traditional Chinese medicine, spatial metabolomics, mass spectrometry imaging, DESI-MSI, MALDI-MSI, SIMS

## Abstract

Spatial metabolomics is an emerging technology that integrates mass spectrometry imaging (MSI) with metabolomics, offering a novel visual perspective for traditional metabolomics analysis. This technology enables in-depth analysis in three dimensions: qualitative, quantitative, and localization of metabolites. Spatial metabolomics precisely reflects the characteristics of metabolic network changes in metabolites within entire tissues or specific micro-regions. It provides a detailed understanding of the pharmacodynamic material basis and mechanisms of action. These capabilities suggest that spatial metabolomics can offer significant technical support for studying the complex pathophysiology of mental disorders. Although the mechanisms underlying mental disorders have been reviewed multiple times, this paper provides a comprehensive comparison between traditional metabolomics and spatial metabolomics. It also summarizes the latest progress and challenges of applying spatial metabolomics to the study of mental disorders and traditional Chinese medicine.

## 1 Introduction

Mental disorders are common and burdensome. Among people with severe mental illness, deaths from unnatural causes have increased significantly, occurring 13 times more frequently compared to the general population, with suicide being the leading cause (20 times higher) (Revier et al., 2015). Antidepressants and antipsychotics remain the primary strategies for treating psychiatric symptoms. However, the long-term efficacy of drug treatment has been questioned. For many individuals with mental illness, antipsychotics do not result in clinically meaningful long-term improvements and often cause significant side effects (Malhi and Mann, 2018), such as weight gain, elevated blood sugar levels, elevated blood lipids, and loss of sexual interest. These side effects frequently lead to withdrawal and discomfort (De Hert et al., 2011; Vancampfort et al., 2015). Over the past few decades, the use of traditional Chinese medicine (TCM) to treat various mental disorders, including depression, has grown significantly. Studies suggest that TCM is a safer alternative to drug therapy, with a lower risk of side effects (Yeung K. S. et al., 2018).

Traditional Chinese medicine operates under the guidance of traditional Chinese medicine theory. It exhibits characteristics such as multi-metabolite, multi-target, multi-approach, and holistic concept (Ma et al., 2023; Wang and Zhang, 2017; Zheng et al., 2024; Song et al., 2023). While traditional Chinese medicine demonstrates remarkable clinical efficacy, its modern development faces constraints due to unclear efficacy substances and mechanisms of action. In recent years, there has been rapid development in metabolomics technology (Misra, 2018; Bingol and Brüschweiler, 2017). Its research strategy, which is based on detecting the dynamic changes of global metabolites, aligns with traditional Chinese medicine theory (Zhang et al., 2010; Wei et al., 2024). This alignment presents new opportunities to address the developmental challenges faced by traditional Chinese medicine. Metabolomics technology has been extensively employed in researching the material basis and pharmacodynamic mechanisms of traditional Chinese medicine, yielding promising results (Wang et al., 2017).

However, the metabolism and distribution of traditional Chinese medicine metabolites in organisms often exhibit precise spatial positioning (Bai et al., 2022). The efficacy of the medication is closely linked to the spatial distribution of biological tissue or micro-regions. Nevertheless, traditional metabolomics methods have limitations in sample pre-processing, resulting in the absence of spatial distribution information of metabolites in tissues (Liu G. X. et al., 2023). This absence poses challenges in fully and objectively interpreting the sites of action and pharmacodynamic mechanisms of traditional Chinese medicine. Spatial metabolomics enables the correlation of metabolites and their biological functions with the anatomical characteristics of biological tissues (Sun et al., 2019; Nakabayashi et al., 2021). This approach allows for a more accurate and scientific analysis of the pharmacodynamic metabolites of traditional Chinese medicines and the regulatory mechanisms of diseases within organisms.

Indeed, these methods also facilitate the comprehensive characterization of metabolic functions at physiological and pathological time scales with high spatial resolution. Mass spectrometry imaging (MSI) is a powerful method to perform *in situ* analysis of the molecular composition of biological tissues while retaining spatial information (Parrot et al., 2018). Furthermore, spatial metabolic characterization holds significant relevance to our comprehension of normal physiological processes and the neuropathological manifestations of neurological disorders (Wang et al., 2022).

Hence, spatial metabolomics technology was utilized to establish the relationship of “molecular structure-spatial distribution-content change-metabolic pathway,” offering novel insights into the search for medicinal metabolites, therapeutic targets, and mechanisms of action of traditional Chinese medicine (Zhao et al., 2023a).

This paper provides a comprehensive overview of the research progress in spatial metabolomics technology concerning the quality control, metabolic distribution, pharmacodynamic mechanisms, and toxicity mechanisms of Chinese medicine. Additionally, it critically examines the limitations and future development directions of spatial metabolomics in the study of Chinese medicine for treating mental diseases. These insights aim to furnish a theoretical basis for advancing the modernization and internationalization of Chinese medicine in the treatment of mental diseases.

## 2 Traditional metabolomics and spatial metabolomics

Metabolomics involves the systematic study of small and medium molecules in biological fluids. The term “metabolomics” was first coined by Dr. Nicholson of Imperial College London in 1999 (Yu et al., 2017). While metabolomic analysis shares similarities with other high-throughput methods like genome sequencing, its rapid response to both exogenous and endogenous stimuli renders it particularly sensitive to changes in health status (Dona et al., 2016). Spatial metabolomics has been developed based on mass spectrometry imaging technology, characterized by its lack of labeling, matrix, and short analysis cycle (McDonnell and Heeren, 2007; Zang et al., 2021). Serving as a novel molecular imaging technology, spatial metabolomics can directly provide spatial distribution information of numerous known or unknown endogenous metabolites and exogenous drugs from biological tissues (Wang Z. et al., 2021). By employing mass spectrometry imaging technology, spatial metabolomics enables the analysis of metabolites in different tissues and organs in three dimensions, including qualitative, quantitative, and localization aspects. This breakthrough overcomes the limitations of traditional metabolomics research, which often loses spatial information. The comparative analysis of traditional and spatial metabolomics platforms and their respective application conditions are summarized in Table 1.

**TABLE 1:** Traditional metabolomics and spatial metabolomics.

Metabolomics	Methods	Resolution	Sample preparation	Advantage	Disadvantage	Application
Traditional metabolomics	Nuclear magnetic resonance (NMR)	Less (Mandal et al., 2024)	Ease (Zinniel et al., 2012; Asampille et al., 2020)	small samples; no sample pretreatment; and nondestructive and noninvasive detection; high reproducibility; relatively fast measurement; sample preparation automation; the sample can be recovered and stored for a long time; quantitative analysis; (Yu et al., 2017; Emwas et al., 2019)	low sensitivity; low spectral resolution; peak overlaps; the number of detectable metabolites is usually less than 200 metabolites; (Emwas et al., 2019; Serkova and Brown, 2012)	nonselective analysis; real-time metabolite profiling of living cells; real-time metabolic flux analysis; (Emwas et al., 2019)
	Gas chromatography-mass spectrometry (GC-MS)	Depends on mass analyzer (Mandal et al., 2024)	Derivatization (Zinniel et al., 2012; Asampille et al., 2020)	non-selective nature; has the advantage over LC/MS/MS of inherently better chromatographic resolution; reproducible; cheap price; mature technology; (Krone et al., 2010; Zeki Ö et al., 2020)	cannot be used to analyse living samples; derivatization; time consuming; not all molecules can be efficiently detected; (Emwas et al., 2019; Zeki Ö et al., 2020)	appropriate to analyze small molecules, thermally stable, volatile, and easily gasified compounds; (Yu et al., 2017)
	Liquid chromatography-mass spectrometry (LC-MS)	Less (Mandal et al., 2024)	Isolation, purification, Preconcentration (Pérez-Fernández et al., 2017)	rapid specific analysis of a limited number of compounds at high sensitivity and it is relatively easily automated; Hydrolysis of conjugates and chemical derivatization are not required; short time consuming and partially automated; (Krone et al., 2010; Dai and Shen, 2022)	cannot be used to analyse living samples; more cost; technology is not mature enough; (Emwas et al., 2019; Dai and Shen, 2022)	analyze the compounds more polar, higher relative molecular mass and lower thermal stability; (Yu et al., 2017)
Spatial metabolomics	DESI-MSI	10–500 um (Zheng et al., 2023; Zhao et al., 2024)	Minimum, frozen tissue (Wang et al., 2017; He et al., 2022a)	the sample preparation process is simple; high efficiency; high throughput; high accuracy; the operating condition of atmospheric pressure; non-destructive; high specificity; quick results; (Zheng et al., 2023; He et al., 2022a; Mi et al., 2020; Tomalty et al., 2023; Calligaris et al., 2015; Chen et al., 2023a)	low spatial resolution; low sensitivity; not suitable for large tissue samples; (Zheng et al., 2023)	small molecules (metabolites, lipids); <20 kDa proteins; (Zheng et al., 2023; Yang et al., 2022)
	MALDI-MSI	1–200um (Zheng et al., 2023; Zhao et al., 2024)	Matrix deposition, freeze fracture and drying, (Wang et al., 2017; Parrot et al., 2018)	high spatial resolution; simplicity of the tissue preparation; high sensitivity; label-free; reliable results; (Zheng et al., 2023; He et al., 2022a; Chen et al., 2023a; Susniak et al., 2020; Mamun et al., 2023; Veerasammy et al., 2020)	the existence of matrix effects; the operating condition of high vacuum; not suitable for large tissue samples; poor sensitivity for some classes of molecules or limited specificity; (Zheng et al., 2023; Zhou et al., 2021)	biological micromolecules (proteins, peptides, lipids); small molecules; glycans; (Zheng et al., 2023; Zhou et al., 2021)
	AFADESI-MSI	40–100 um (Zheng et al., 2023; Zhao et al., 2024)	Freeze fracture and drying (He et al., 2022a; Zhou et al., 2024a; Shen et al., 2023; Luo et al., 2018)	wide range in slice size; the operating condition of atmospheric pressure; minimum sample preparations; improve sensitivity and spatial resolution from DESI; large coverage; wide field; (Zheng et al., 2023; He et al., 2022a)	not suitable for macromolecules (proteins, peptides); low reproducibility of results due to complex parameters; (Zheng et al., 2023; He et al., 2022a)	small molecule (lipids, small molecules below 500da); (Zheng et al., 2023; He et al., 2022a)
	SIMS-MSI	0.05–0.5 um (Zheng et al., 2023; Zhao et al., 2024)	Freeze fracture and drying (Wang et al., 2017)	high spatial resolution; high efficiency; subcellular imaging; high sensitivity; high selectivity; high dynamic range; (Zheng et al., 2023; Chen et al., 2023a; Shen et al., 2024)	the ion beam may cause the fragmentaion and the damage of the surface of the sample; complexity of datasets; (Zheng et al., 2023; Shen et al., 2024)	wide range; self-assembly monolayer characterization; solid-electrolyte interphase analysis; single cell chemical imaging; environmental related particle investigation; (Zheng et al., 2023; Huang et al., 2017)

### 2.1 Desorption electrospray ionization mass spectrometry (DESI-MSI)

DESI-MS, introduced in 2004, is an atmospheric pressure environmental ionization method that directly ionizes solid-phase samples (Takáts et al., 2004). DESI-MSI employs the fundamental principle of electrospray ionization, wherein solvent droplets are rasterized and desorbed directly onto the sample surface (Parrot et al., 2018; Eberlin et al., 2011). DESI operates at room temperature, eliminating the need for freeze-drying prior to analysis. This method of tissue imaging minimizes sample damage through environmental ionization mass spectrometry, enabling repeated measurements of samples from diverse biological sources (Soudah et al., 2023). Ambient MSI offers a user-friendly interface and facilitates the rapid analysis of larger samples, thereby facilitating real-time diagnostic capabilities (Luo et al., 2013; Keller et al., 2018).

However, enhancing the sensitivity of DESI-MSI presents substantial challenges (Wang et al., 2017). Recent studies have demonstrated that the sensitivity and specificity of DESI-MSI nanoparticles can be enhanced by incorporating silver ions into the solvents (Lillja and Lanekoff, 2022). Researchers have developed a compact post-photoionization module integrated with DESI, enabling the detection of enhanced signal strength for non-polar compounds. This advancement significantly enhances the sensitivity of DESI-MSI to non-polar compounds (Liu C. et al., 2019). Furthermore, there are emerging indications that the spatial resolution of DESI will pose a substantial impediment in numerous other applications where it could be potentially beneficial (Qi et al., 2021). Consequently, scientists are currently engaged in a concerted effort to significantly enhance the spatial resolution of DESI. Subsequent research has demonstrated that nano DESI-MSI possesses the potential to attain even finer spatial resolution, potentially reaching a resolution of 10 microns (Yin et al., 2018; Yin et al., 2019; Yang et al., 2023).

### 2.2 Matrix-assisted laser desorption/ionization mass spectrometry imaging (MALDI-MSI)

The concept of MALDI-MSI was initially introduced in the early 2000s (Morisasa et al., 2019). MALDI is a soft ionization technique that involves the co-crystallization of a sample molecule or analyte with a matrix to form a sample matrix crystal. The matrix functions as a proton donor or acceptor, ionizing the analyte (Yalcin and de la Monte, 2015). MALDI-MSI operates by directing a laser beam at the surface of a specimen, typically a frozen section of tissue. This laser action induces the desorption of ions from the tissue, which are subsequently analyzed through a mass spectrometer (Basu and Agar, 2021; Kuik et al., 2024). Despite ongoing technological advancements, the low detection sensitivity of certain compounds poses a significant challenge that requires effective solutions. Research has indicated that poor sensitivity is often associated with reduced ionization efficiency, low analyte and matrix ion abundance, or endogenous interference. Histochemical derivatization has emerged as a crucial strategy to address these challenges, preserve tissue integrity, and mitigate potential dislocations (Merdas et al., 2021).

### 2.3 Airflow-assisted desorption and electrospray ionization mass spectrometry imaging (AFADESI-MSI)

AFADESI-MSI employs DESI technology to directly ionize the sample using an electrospray plume. Subsequently, a gas stream propels the ions over extended distances, enabling mass spectrometry imaging (Luo et al., 2013). In addition to inheriting the advantages of DESI-MSI, AFADESI-MSI can also attain exceptionally high metabolite coverage. It is an environmental molecular imaging technology characterized by its high sensitivity, extensive coverage, and exceptional chemical specificity (He M. J. et al., 2022). A significant advantage of this approach is its direct predictive applicability to a substantial number of candidate metabolites and metabolic enzymes, eliminating the necessity to define a specific target of interest beforehand (Sun et al., 2019). Although this novel technique yields drug signal strength, it cannot objectively reflect the absolute drug content in various tissues due to sample heterogeneity, ion inhibition, analyte extraction efficiency, and ionization efficiency (Zhang et al., 2020).

### 2.4 Secondary ion mass spectrometry (SIMS-MSI)

The SIMS instrument bombards the sample surface with a finely focused primary ion beam (an analysis gun) to generate characteristic secondary ions from the sample surface. These secondary ions are subsequently detected using a mass analyzer. By rasterizing the primary ion beam on the surface of a solid sample, mass-resolved secondary ion images can be obtained, thereby providing chemical mapping of each component of the surface (Huang et al., 2017). The primary advantage of SIMS lies in its capability to measure the spatial localization of molecules with exceptional spatial resolution. It is particularly effective in targeting inorganic compounds or biomolecules with relatively low molecular weights (Wu et al., 2013). Although samples for SIMS do not necessitate any special surface treatment, it is important to note that SIMS can be a destructive analysis technique, which may lead to sample loss. Furthermore, quantifying the composition of SIMS samples can be challenging due to matrix effects and fragmentation processes that occur during SIMS analysis (Huang et al., 2017).

In summary, mass spectrometry imaging (MSI), a tool capable of *in situ* quantitative qualitative and two-dimensional imaging, is characterized by high stability, high throughput, and label-free. The above MSI techniques have their characteristics. MALDI is suitable for detecting various small and large molecules, and it is the most used technique in multiple fields, but the preparation process is relatively complicated. DESI is more accurate for the *in situ* localization of small molecules in tissue slices, but the spatial resolution is relatively low compared with the other techniques. DESI has a broader range of application scenarios than the different techniques, and it can be used for detection at room temperature. SIMS can measure the spatial localization of molecules with high spatial resolution, but the sample components used for SIMS may be lost, generating fragment ions that can severely interfere with the detection signals of small chemical molecules. AFADESI directly inherits the advantages of DESI but also optimizes the technology based on it.

## 3 Spatial metabolomics and Chinese medicine

### 3.1 Quality control

Chinese medicine quality control plays a vital role in ensuring the clinical efficacy of Chinese medicine (Li X. R. et al., 2021). The medicinal parts, metabolites, and distribution of traditional Chinese medicine directly reflect its quality, but traditional analysis methods often face challenges in achieving comprehensive assessments. MSI emerges as a novel analytical method that overcomes the technical limitations of traditional approaches. MSI technology encompasses secondary ionization (SI), matrix-assisted laser desorption ionization (MALDI), and desorption electrospray ionization (DESI) methods based on ionization techniques (Ganesana et al., 2017). Notably, MSI eliminates the need for intricate sample pretreatment steps and offers the capability to detect known or unknown metabolites with high throughput, sensitivity, and resolution (Zheng et al., 2023). It serves as a straightforward and swift approach for identifying quality markers in Chinese medicine, enabling the direct characterization of chemical features and spatial distribution across various samples. Consequently, MSI holds promising applications in Chinese medicine quality control (Jiang H. et al., 2022; Dong and Aharoni, 2022). Table 2 presents an overview of spatial metabolomics studies in Chinese medicine quality control.

**TABLE 2:** Spatial metabolomics and quality control.

Drug	Medicinal parts	Analytical technique	Characterization metabolite	References
Panax quinquefolius L. [Araliaceae]	Root	UPLC-Q-TOF/MS, DESI-MSI	Ginsenoside Rg1, malonyl-ginsenoside Rc, ginsenoside Ro, malonyl-ginsenoside Rd	Luo et al. (2024)
Salvia miltiorrhiza Bunge [Lamiaceae]	Root	DESI-MSI	Phenolic acids, flavonoids, tanshinones, carbohydrates, lipids	Tong et al. (2022)
Phyllanthus emblica L. [Phyllanthaceae]. Fruit (PEF)	PEF surface white frost	UPLC-Q-TOF-MS/MS, DESI-MSI	Organic acids, fatty acids, tannins	Lin et al. (2024)
Glycyrrhiza uralensis Fisch. ex DC. [Fabaceae]	Root	high-resolution liquid chromatography/mass spectrometry, DESI-MSI	Flavonoids, triterpenoids	Zhao et al. (2023b)
Ligustrum lucidum W.T.Aiton [Oleaceae]	Fruit	UHPLC/Q-Orbitrap-MS, MALDI-MSI	10-hydroxyoleoside dimethylester, 8-demethyl-7-ketoliganin, elenolic acid, salidroside, neonuezhenide/isomer, verbascoside/isomer, luteoline, nuzhenal A	Li et al. (2020a)
Panax notoginseng (Burkill) F.H.Chen [Araliaceae]	Fresh root	UPLC-QTOF-MS, MALDI-MS	Ginsenosides	Fan et al. (2022)
Scutellaria baicalensis Georgi [Lamiaceae]	Root, stem, leaf	MALDI-MSI	Flavonoids, glycosides	Zhou et al. (2024b)
Ganoderma [Ganodermataceae]	Fruiting body	LC-MS, DESI-MSI	Triterpenoids, fatty acids	Xia et al. (2024)
Salvia miltiorrhiza Bunge [Lamiaceae]	Root, stem, leaf	MALDI-MSI	Amino acids, phenolic acids, fatty acids, oligosaccharides, cholines, polyamines, tanshinones, phospholipids	Sun et al. (2020)
Tripterygium wilfordii Hook.f. [Celastraceae]	Root	MALDI-MSI	Triterpenoids, sesquiterpene alkaloids	Lange et al. (2017)
Paeonia lactiflora Pall. [Paeoniaceae]	Root	AP-SMALDI MSI	Gallotannins, monoterpene glucosides	Li et al. (2016)
Putterlickia verrucosa (E.Mey. ex Harv. and Sond.) Sim [Celastraceae]	Rhizome	AP-SMALDI MSI	Maytansinoids	Eckelmann et al. (2016)
Glycyrrhiza glabra L. [Fabaceae]	Rhizome	AP-MALDI-MSI	Flavonoids, flavonoid glycosides, saponins	Li et al. (2014)
Ginkgo biloba L. [Ginkgoaceae]	Leaf	AP-MALDI-MSI	Flavonoid glycosides, biflavonoids	Beck and Stengel (2016)
Panax ginseng C.A.Mey. [Araliaceae]	Root	MALDI-MSI, DESI-MSI	Ginsenosides in Panax ginseng with different age, ginsenosides localization in Panax ginseng root	Bai et al. (2016), Lee et al. (2017), Taira et al. (2010), Yang et al. (2021)
Panax ginseng C.A.Mey. [Araliaceae], Panax quinquefolius L. [Araliaceae], Panax notoginseng (Burkill) F.H.Chen [Araliaceae]	Root	MALDI-MSI	Saponins	Wang et al. (2016)
Aconitum carmichaelii Debeaux [Ranunculaceae]	Lateral roots	MALDI-MSI	Aconitum alkaloids	Wang et al. (2009)
Paeonia × suffruticosa Andrews [Paeoniaceae], Paeonia lactiflora Pall. [Paeoniaceae]	Root	MALDI-MSI	Monoterpene and paeonol glycosides, tannins, flavonoids, saccharides, lipids	Li et al. (2021b)
Salvia miltiorrhiza Bunge [Lamiaceae]	Root, stem, leaf	MALDI-MSI	Differential distribution of salvianolic acids and tanshinones	Li et al. (2020b)
Scutellaria baicalensis Georgi [Lamiaceae]	Root	PALDI-MSI	Baicalein and wogonin, mainly were distributed in the epidermis of the root	Feng et al. (2014)
Isatis tinctoria subsp. tinctoria [Brassicaceae]	Root	Q-TOF/MS, DESI-MSI	Alkaloids, sulfur-containing compounds, phenylpropanoids, nucleosides, amino acids, organic acids, flavonoids, phenols, terpenes, saccharides, peptides, sphingolipids	Nie et al. (2022a)
Cordyceps sinensis	Caterpillar	SIMS-MSI	Fatty acids, glycerides, glycerophospholipids, amino acids, nucleosides, monosaccharides, sphingolipids, sterols	Liu et al. (2022a)
Curcuma longa L. [Zingiberaceae]	Root	AP-MALDI-MSI	Curcumin	Shimma and Sagawa (2019)
Panax notoginseng (Burkill) F.H.Chen [Araliaceae]	Root, rhizome	MALDI-MSI	Notoginsenosides, ginsenosides, amino acids, dencichine, gluconic acid, low-molecular-weight organic acids, dencichine, arginine, glutamine	Sun et al. (2021)
Coptis chinensis Franch. [Ranunculaceae]	Rhizome	UPLC-QQQ-MS/MS, SIMS-MSI	Berberine, epiberberine, coptisine, palmatine, columbamine, jatrorrhizine, tetrahydricheilanthifolinium, oxyberberine	He et al. (2022b)
Dendrobium nobile Lindl. [Orchidaceae]	Stem	UPLC-QTOF-MS, MALDI-MSI	Alkaloids, sesquiterpenoids	Liu et al. (2022b)
Ginkgo biloba L. [Ginkgoaceae]	Root, stem, leaf	FT-ICR MS, MALDI-MSI	Flavonoids, saccharides, phospholipids, chlorophylls, ginkgolides	Li et al. (2018)
Panax ginseng C.A.Mey. [Araliaceae]	Fresh root	LACFI-MSI	Monacylglycerides, diacylglycerides, triacylglycerides, organic acids, ginsenosides	Lu et al. (2023)
Lycium barbarum L. [Solanaceae]	Fruit	MALDI-MSI	Choline, betaine, citric acid, hexose, sucrose, phenolic acids, flavonoids	Zhao et al. (2021)
Forsythia suspensa (Thunb.) Vahl [Oleaceae]	Fruit	MALDI-MSI	Pinoresinol, phillygenin, forsythoside A, forsythoside E, rutin, caffeic acid, malic acid, citric acid, stearic acid, oleic acid, linoleic acid	Jing et al. (2022)
Salvia miltiorrhiza Bunge [Lamiaceae]	Root	MALDI-MSI	Tanshinones, salvianolic acids, polyamines, phenolic acids, amino acids, oligosaccharides	Sun et al. (2022)
Aconitum carmichaelii Debeaux [Ranunculaceae]	Fresh root	DESI-MSI	Alkaloids	Liu et al. (2022c)
Gastrodia elata Blume [Orchidaceae]	Rhizome	MALDI-MSI	Parishins, hydrolases,	Ma et al. (2022)
Salvia miltiorrhiza Bunge [Lamiaceae]	Root	LC-MS/MS, DESI-MSI	Diterpenoids	Xia et al. (2023)
Panax notoginseng (Burkill) F.H.Chen [Araliaceae]	Nodule	MALDI-MSI	Ginsenoside	Yu et al. (2024)
Aconitum pendulum N.Busch [Ranunculaceae]	Dry roots	HPLC -QqQ-MS, DESI-MSI	Alkaloids	Tan et al. (2023)
Reynoutria multiflora (Thunb.) Moldenke [Polygonaceae]	Root	UPLC-QTOF MS, DESI-MSI	Stilbenes, flavonoids, anthraquinones, alkaloids, naphthalenes	Cai et al. (2023)
Paeonia lactiflora Pall. [Paeoniaceae]	Fresh roots	DESI-MSI	Paeoniflorin, benzoylpaeoniflorin, oxypaeoniflorin, gallic acid, 1,2,3,4,6-pentagalloylglucose, albiflorin, catechin	Chen et al. (2022a)
Isatis tinctoria L. [Brassicaceae]	Dried root	AP-MALDI-MSI	Alkaloid, organic acids, peptides, saccharides, flavonoids, aromatics	Nie et al. (2021)
Panax quinquefolius L. [Araliaceae]	Multi-steamed roots	UPLC-Q-TOF-MS/MS, MALDI-MSI	Ginsenosides	Li et al. (2024a)
Angelica dahurica (Hoffm.) Benth. and Hook.f. ex Franch. and Sav. [Apiaceae]	Root	HPLC, DESI-MSI	Coumarins	Wu et al. (2023)
Clausena lansium (Lour.) Skeels [Rutaceae]	Root, stem, leaf, seed	MALDI-MSI	Active alkaloids, coumarins, sugars, organic acids	Tang et al. (2021)
Paris yunnanensis Franch. [Melanthiaceae]	Rhizome	MALDI-MSI	Steroidal saponins, amino acids, organic acids, phytosterols, phytoecdysones, nucleosides, esters	Zhang et al. (2022a)
Panax bipinnatifidus var. bipinnatifidus [Araliaceae]	Rhizome	UHPLC/QTOF-MS, DESI-MSI	Ginsenoside	Jiang et al. (2023)
Cordyceps cicadae	Sclerotium, coremium	Q-TOF/MS, DESI-MSI	Nucleosides, amino acids, polysaccharides, organic acids, fatty acids	Cao et al. (2024)
Lepidium meyenii Walp. [Brassicaceae]	Root	MALDI-MSI	Imidazole alkaloids	Mi et al. (2020)
Aconitum napellus L. [Ranunculaceae]	Root	UHPLC-QTOF-MS, DESI-MSI	Aconitum alkaloids	Ren et al. (2023)
Angelica dahurica (Hoffm.) Benth. and Hook.f. ex Franch. and Sav. [Apiaceae]	Root	MALDI-MSI	Coumarins	Gao and Li (2023)
Curcuma longa L. [Zingiberaceae]	Root	MALDI-MSI	Curcumin	Nie et al. (2022b)
Pueraria montana var. lobata (Willd.) Maesen and S.M.Almeida ex Sanjappa & Predeep [Fabaceae]	Dried roots	LC-MS, AFADESI-MSI	Saccharide, vitamin, inosine, 3′-hydroxyl puerarin	Guo et al. (2023)
Paeonia lactiflora Pall. [Paeoniaceae]	Root	UPLC, DESI-MSI	Albiflorin, catechin, Paeoniflorin, benzoylpaeoniflorin, oxypaeoniflorin, gallic acid1, 2,3,4,6-pentagalloylglucose	Chen et al. (2022b)
Shaoyao Gancao Decoction	Decoction	UHPLC-DAD, DESI-MSI	Paeoniflorin, liquiritin, glycyrrhizic acid, albiflorin, licoricesaponin G2, licoricesaponin J2, gallic acid, citric acid, p-hydroxybenzoic acid	Qu et al. (2020)
Paris yunnanensis Franch. [Melanthiaceae]	Rhizome	MALDI-MSI	steroidal saponins, amino acids, organic acids, phytosterols, phytoecdysones, nucleosides, esters	Zhang et al. (2022b)
Panax quinquefolius L. [Araliaceae]	Root	UPLC-Q-TOF-MS, MALDI-MSI	ginsenosides	Li et al. (2024b)
Aconitum carmichaelii Debeaux [Ranunculaceae]	Different growth stages and different parts	UPLC-Q-TOF-MS, DESI-MSI	Paclobutrazol	Hou et al. (2023)
Angelica pubescens Maxim. [Apiaceae]	Fresh roots	MALDI-MSI	Coumarins	Li et al. (2023a)
Panax ginseng C.A.Mey. [Araliaceae]	Root	DESI-MSI	Ginsenosides, lipids	Wang et al. (2024a)
Scutellaria baicalensis Georgi [Lamiaceae]	Root	LD-DBDI-MSI	Anthraquinone	Xiao et al. (2024)
Gynochthodes officinalis (F.C.How) Razafim. and B.Bremer [Rubiaceae]	Root	MALDI-MSI	Iridoid, saccharous	Qiao et al. (2022)
Aconitum carmichaelii Debeaux [Ranunculaceae]	Root	MALDI-MSI	Alkaloids	Dai et al. (2022)
Panax ginseng C.A.Mey. [Araliaceae], Panax quinquefolius L. [Araliaceae], Panax notoginseng (Burkill) F.H.Chen [Araliaceae]	Root	DESI-MSI	Saponins, acid-hydrolyzed oligosaccharides	Wang (2022)
Isatis tinctoria L. [Brassicaceae]	Root	MALDI-MSI, DESI-MSI	More than 100 components	Nie et al. (2023)
*Nelumbo nucifera* Gaertn. [Nelumbonaceae]	Seed	MALDI-MSI	Alkaloids, flavonoids, amino acids, fatty acids, organic acids, cholines, phospholipids	Sun et al. (2021)
Panax notoginseng (Burkill) F.H.Chen [Araliaceae]	Root	MALDI-MSI	Notoginseng saponins	Liu et al. (2020)
Panax ginseng C.A.Mey. [Araliaceae], Panax notoginseng (Burkill) F.H.Chen [Araliaceae], Panax quinquefolius L. [Araliaceae]	Root	MALDI-MSI	Saponins	Bai (2016)
Rauvolfia tetraphylla L. [Apocynaceae]	Stem, leaf, root, fruit	DESI-MSI	Indole alkaloids	Mohana Kumara et al. (2019)
Cannabis sativa L. [Cannabaceae]	Leaf	MALDI-MSI, DESI-MSI	Cannabinoids	Lorensen et al. (2023a)
Salvia divinorum Epling and Játiva [Lamiaceae]	Leaf	DESI-MSI	Salvinorin A	Kennedy and Wiseman (2010)
Rauvolfia tetraphylla L. [Apocynaceae]	Leaf	MALDI-MSI, DESI-MSI	Monoterpenoid indole alkaloids	Lorensen et al. (2023b)
Citrus × aurantium L. [Rutaceae]	Peel	DESI-MSI	Polar compounds	Bagatela et al. (2015)
Angelica decursiva (Miq.) Franch. and Sav. [Apiaceae]	Root	MALDI-MSI	Coumarin	Li and Li (2024)

UPLC-Q-TOF/MS, ultra-performance liquid chromatography quadrupole/time of flight-mass spectrometry. UPLC-Q-TOF-MS/MS, ultra-performance liquid chromatography quadrupole time-of-flight mass spectrometry. UHPLC/Q-Orbitrap-MS, ultra-high performance liquid chromatography/quadrupole-Orbitrap mass spectrometry. AP-SMALDI MSI, atmospheric-pressure scanning microprobe matrix-assisted laser desorption/ionization mass spectrometry imaging. AP-MALDI-MSI, atmospheric-pressure matrix-assisted laser desorption/ionization mass spectrometry imaging. PALDI-MSI, plasma assisted laser desorption ionization mass spectrometry. Q-TOF/MS, quadrupole-time-of-flight mass spectrometry. UPLC-QQQ-MS/MS, ultra-high-performance liquid chromatography coupled with triple quadrupole mass spectrometry. SIMS, secondary ion mass spectrum imaging. FT-ICR MS, Fourier-transform ion cyclotron resonance mass spectrometry. LACFI-MSI, laser ablation carbon fiber ionization MSI. HPLC, high-performance liquid chromatography. HPLC-QqQ-MS, high-performance liquid chromatography-tandem triple quadrupole mass spectrometry. UHPLC/QTOF-MS, ultra-high performance liquid chromatography/quadrupole time-of-flight mass spectrometry. AFADESI-MSI, air flow-assisted desorption electrospray ionization-mass spectrometry imaging. UHPLC-DAD, ultra-high performance liquid chromatography with diode array detection. LD-DBDI-MSI, laser desorption-dielectric barrier discharge ionization MSI.

### 3.2 Spatial distribution and pharmacodynamic mechanism

The distribution and metabolism of TCM active metabolites in tissues are crucial for identifying target organs, understanding the pharmacodynamic material basis, and evaluating potential adverse reactions of TCM. However, traditional analysis techniques often destroy tissue structure during sample preparation, making it difficult to clearly characterize the regional distribution of active ingredients and metabolites of TCM. MSI can extract extensive data and provide information about the spatial distribution of these data by analyzing tissue slices (Xu et al., 2022). Spatial metabolomics can simultaneously characterize the spatial metabolic distribution of TCM active metabolites and their metabolites in the whole or micro-regions of different tissues and organs. This approach presents a more complete metabolic process and is a significant analytical technique in neuroscience research (Liang et al., 2022). Table 3 shows studies on the metabolic distribution of TCM in organisms.

**TABLE 3:** Spatial metabolome, spatial distribution and pharmacodynamic mechanism.

Medicine	Disease	Dose administration	Sample	Analytical technique	Apatial distribution, pharmacodynamic mechanism
Ginseng and American ginseng (Huang et al., 2023)	—	Gavage	Rat brain	DESI-MSI	A total of 25 neurochemicals were imaged and identified in brain section. 17 neurochemicals were classified as warm markers. 8 neurochemicals were identified as cool markers, correlated with the cool properties of American ginseng
Fritillariae Cirrhosae Bulbus (Qin et al., 2024)	Pulmonary fibrosis	Gavage	Rat lung tissue	DESI-MSI	The content of L-arginine in the fibrotic regions of lung tissues in rats with pulmonary fibrosis exhibited significant differences compared to the model group rats. the phosphatidylcholine content in the fibrotic regions of the lung tissues was lower than that in the model group rats
Pterostilbene (Ban et al., 2024)	Cerebral ischemia/reperfusion injury	Gavage	Rat brain	AFADESI-MSI	Pterostilbene was widely and abundantly distributed in ischemic brain tissue
Scutellarin and its metabolites (Wang et al., 2021b)	—	Ip injection	Mouse kidney tissues	MALDI-MSI	Scutellarin and scutellarein were found to be located in the cortex and medulla regions of the kidney with relatively high abundance, whereas the remaining metabolites appeared in the cortex with low abundance
Rhodiola crenulata (Hou et al., 2024)	High-altitude hypoxic brain injury	Gavage	Mice brain tissue	UHPLC-MS, MALDI-MSI	Glutathione level was markedly lowered in the HH group compared with the control group, while RCE (R. crenulate extract) and Sal (salidroside) treatment corrected this aberrant decrease after HHBI.
Notoginseng leaf triterpenes (PNGL) (Wang et al., 2021c)	Cerebral ischemia/reperfusion injury	Intraperitoneal injection	Rat brain tissue	MALDI-MSI	PNGL can significantly decreased the content of glucose and citric acid in both the striatum and cerebral cortex
Uncaria alkaloids (Gao et al., 2022)	—	—	Rat brain tissue	DESI-MSI	The distribution trend of different Uncaria alkaloids in the rat brain was listed as monoterpene indole alkaloids > monoterpene oxindole alkaloids, R-configuration epimers > S-configuration epimers
Radix Aconiti Lateralis Preparata Extracts (Wu et al., 2019)	Myocardial infarction	Intragastrically administered	Rat heart	MALDI-MSI	Radix Aconiti Lateralis Preparata extract (RAE) and fuzi total alkaloid (FTA) significantly improved left ventricular function and structure, and reduced myocardial damage and infarct size in rats with myocardial infarction by the left anterior descending artery ligation
notoginsenoside R1 (NG-R1) (Zhu et al., 2020)	Ischemic stroke	Intraperitoneal (i.p.) injection	Rat brain tissue	MALDI-MSI	NG-R1 regulated ATP metabolism, the tricarboxylic acid (TCA) cycle, the malate-aspartate shuttle, antioxidant activity, and the homeostasis of iron and phospholipids in the striatum and hippocampus of middle cerebral artery occlusion/reperfusion (MCAO/R) rats
Xiaoke pills (Zhu et al., 2022)	—	Feeding solution	Zebrafish	UPLC-HRMS, DESI-MSI	A total of 49 compounds related to Xiaoke pills (including 13 prototypical components and 36 metabolites) were detected in zebrafish
ginsenoside Rg1 (Wei et al., 2021)	—	Intravenously administrated	Rats different tissues (heart, liver, spleen, lung, kidney and brain)	LC-MS/MS, DESI-MSI	Rg1 mainly accumulated in the pelvis section of kidney. the imaging result of brain implied that Rg1 might be distributed in the pons and medulla oblongata region of brain at 15 min after intravenous administration
Paclitaxel (PTX) and its prodrug (PTX-R) (Zhang et al., 2020)	Xenograft tumor model	Intravenous route (i.v.) via the tail vein	Mice intact whole-body	VC-QMSI	PTX was widely distributed in multiple organs throughout the dosed body in the PTX-injection group and the PTX-liposome group
XueFu ZhuYu decoction (XFZY) (Li et al., 2021c)	Traumatic brain injury (TBI)	Intragastrically administrated	Rat brain tissue	DESI-MSI	Several phosphatidylcholines, phosphatidylethanolamines, phosphatidic acids, and diacylglycerols were found to be significantly upregulated particularly in midbrain and thalamus after TBI and XFZY treatment
Thymoquinone (Tian et al., 2020)	Cerebral ischemia reperfusion injury	Intraperitoneal injection	Rat brain tissue	MALDI-MSI	Thymoquinone reduced abnormal accumulations of glucose, citric acid, succinate and potassium ions
Shenfu injection (Liu et al., 2019b)	Septic shock	Intravenous injection	Rabbit heart	MALDI-MSI	Shenfu injection can increase the contents of ATP and taurine while reducing the content of AMP in the heart tissue during septic shock
Ferulic acid (Liu et al., 2023b)	Diabetic cardiomyopathy	Intragastric administration	Rat heart	AFADESI-MSI, MALDI-MSI	The repeated oral administration of ferulic acid during 20 weeks significantly improved most of the metabolic disorders in the DCM model
Isosteviol compounds (Ke et al., 2021)	—	Immersion	Zebrafish	DESI-MSI	The signal of STVNa distributed uniformly in zebrafish,but K-9 distributed more concentrate in in specific organs or parts of the eyes,the brain,the pelvic fin,or the caudal fin
Tianyuan Zhitong Prescription (Cheng et al., 2024)	—	Gavage	Mice brain	UPLC-Q-TOF-MS, DESI-MSI	27 brain absorption components (10 organic acids, 5 glycosides, 4 alkaloids, 1 phenol, 4 flavonoids, 2 phthalides and 1 other compound)
Hordenine (Zhou et al., 2024)	—	Gavage	Rats different tissues (heart, kidney, brain, pituitary, spleen)	DESI-MSI	The kidneys exhibit the strongest signal and the most significant distribution changes among all tissues
Taohong Siwu decoction (Wang et al., 2024b)	Ischemic stroke	Gavage	Rat brain	DESI-MSI	30 metabolites exhibited significant dysregulation in the ischemic brain regions, specifically the cortex and striatum, following ischemic injury,
Shuangshen Ningxin Fomula (SSNX) (Li et al., 2024c)	Myocardial ischemia-reperfusion injury	Gavage	Rat heart	MALDI-MSI	The adenosine triphosphate distribution in the ischemic infarction area of the SSNX group increased significantly
Radix ginseng-Schisandra chinensis Herb Couple (Fan et al., 2024)	Alzheimer’s disease	Gavage	Mice brain	AFADESI-MSI	Twenty-eight biomarkers were identified

UPLC-HRMS; ultra-performance liquid chromatography-high-resolution mass spectrometry. VC-QMSI; virtual calibration quantitative mass spectrometry imaging.

### 3.3 Toxicity mechanism

Drug safety poses a significant threat to human health. Toxicological analysis and safety evaluation are crucial aspects of drug development. Conventional analysis methods cannot provide spatial distribution information. However, spatial dimension analysis can supplement safety evaluations, enabling better prediction and assessment of drug toxicity (Chen et al., 2023b). Spatial metabolomics allows us to study the distribution of toxic Chinese medicine components and their metabolites in tissues and organs. This technique provides a scientific basis for identifying toxic target organs and revealing toxic molecular mechanisms (Table 4).

**TABLE 4:** Spatial metabolome and toxicity mechanisms.

Medicine	Toxicity	Dose administration	Sample	Analytical technique	Toxicity mechanisms
Component D of Polygonum multiflorum Thunb (PM-D) (Jiang et al., 2022b)	Hepatotoxicity	Orally administered	Mice liver	AFADESI-MSI	Metabolites such as taurine, taurocholic acid, adenosine, and acyl-carnitines were associated with PM-D-induced liver injury
Aristolochic acids (Wang et al., 2020)	Nephrotoxicity	Orally administered	Rat kidney	AFADESI-MSI	38 metabolites related to the arginine-creatinine metabolic pathway, the urea cycle, the serine synthesis pathway, metabolism of lipids, choline, histamine, lysine, and adenosine triphosphate were significantly changed in the group treated with aristolochic acid I
Rotenone (Li et al., 2023b)	Rotenone toxicity	Leaf disk dipping	Plutella xylostella	MALDI-MSI	Rotenone significantly affected purine and amino acid metabolisms, indicating that adenosine monophosphate and inosine were distributed in the whole body of P. xylostella with elevated levels, while guanosine 5′-monophosphate and tryptophan were significantly downregulated

## 4 Spatial metabolomics and mental disorders

### 4.1 Spatial metabolomics studies on schizophrenia

Schizophrenia, a major mental illness, involves lipids playing a crucial role. The authors (Matsumoto et al., 2011) have demonstrated the association between lipid analysis and brain functional mapping in *postmortem* human brains. They identified the types of lipids in normal human brains using LC/ESI-MS/MS. Subsequently, MALDI-MSI analysis of brain tissue was conducted to screen for differentially expressed lipid types between the control group and two schizophrenia patients. In this study, the authors report the abnormal distribution of a molecular species of phosphatidylcholine (PC), specifically in the cortical layer of the frontal cortex region, *postmortem* in patients with schizophrenia. Additionally, PC (diacyl-16:0/20:4) containing arachidonic acid showed an increase in the frontal cortex of patients with schizophrenia. MALDI-MSI holds a specific advantage in revealing abnormalities in local lipid metabolism in the human brain after death. Moreover, it complements previous findings indicating abnormal brain lipid composition in schizophrenia patients (McNamara et al., 2007; Taha et al., 2013).

The corpus callosum (CC) serves to connect the brain’s hemispheres, yet individuals with schizophrenia exhibit impaired interhemispheric communication, potentially contributing to brain disconnection (Guo et al., 2013). Researchers (Vendramini et al., 2016) utilized DESI-MSI to compare lipid content in *postmortem* CC samples from two schizophrenia patients and two controls in a label-free manner. The findings reveal a noteworthy reduction in the distribution of phosphatidylcholine in patients with schizophrenia. Interestingly, the 760 Da ions show a much lower abundance of phosphatidylcholine compared to the 788 Da ions. This study marks the first investigation into CC white matter in schizophrenia patients and strongly supports the hypothesis that phospholipid dysfunction is prevalent in schizophrenia (Ross et al., 1997). Despite limitations in sample size, these studies contribute to the molecular understanding of the disease, as well as the identification of biomarkers and drug targets. Phospholipids are bioactive substances crucial for brain function. To analyze differences in the amount and type of phospholipids present in the brain tissue of schizophrenic patients, the authors (Matsumoto et al., 2017) conducted a comprehensive analysis of phospholipids in the *postmortem* brains of elderly schizophrenic patients. In LC-ESI/MS/MS, the authors found significantly reduced levels of 16:0/20:4-phosphatidylinositol (PI) in the prefrontal cortex of the brain in patients with schizophrenia, while 16:0/20:4-PI was most notably reduced in the gray matter in MALDI-MSI.

### 4.2 Spatial metabolomics studies on depression

Stress represents a risk factor for the development and exacerbation of various diseases, including neuropsychiatric disorders and depression (Sanacora et al., 2022; Park et al., 2019). The endocannabinoid 2-arachidonoylglycerol (2AG) serves as a vital regulator of stress response, with its brain levels increasing in response to heightened stress. Researchers (Islam et al., 2022) investigated the impact of stress on 2AG levels in specific brain regions of senescence-accelerated mouse prone (SAMP8). Utilizing DESI-MSI, they observed a significant increase in 2AG levels in the hypothalamus, midbrain, and hindbrain of SAMP8 mice following 3 days of water immersion stress. Previous reports (Zhai et al., 2023) utilizing DESI-MSI analysis of coronal brain sections from stressed mice indicated that 2-AG levels were highest in the hypothalamus region and lowest in the hippocampus, spanning from forebrain to cerebellum. Furthermore, this study demonstrated elevated levels of endocannabinoid 2-AG in the Anterior Cingulate Cortex, Caudate Putamen, Nucleus Accumbens, and Piriform Cortex in individuals experiencing chronic stress. *postpartum* depression (PPD) presents a severe mental disorder with significant adverse effects on maternal health. Researchers (Sheng et al., 2024) employed MSI and targeted metabolomics analysis to investigate metabolic changes in the brains of *postpartum* mice with GABA_A_R Delta-subunit defects (Gabrd^−/−^), serving as a specific preclinical model of PPD. This study identified the downregulation of prostaglandin D2 (PGD2) in the central amygdala (CeA) as the most notable change in PPD.

### 4.3 Spatial metabolomics studies on drug addiction

**FIGURE 1:**
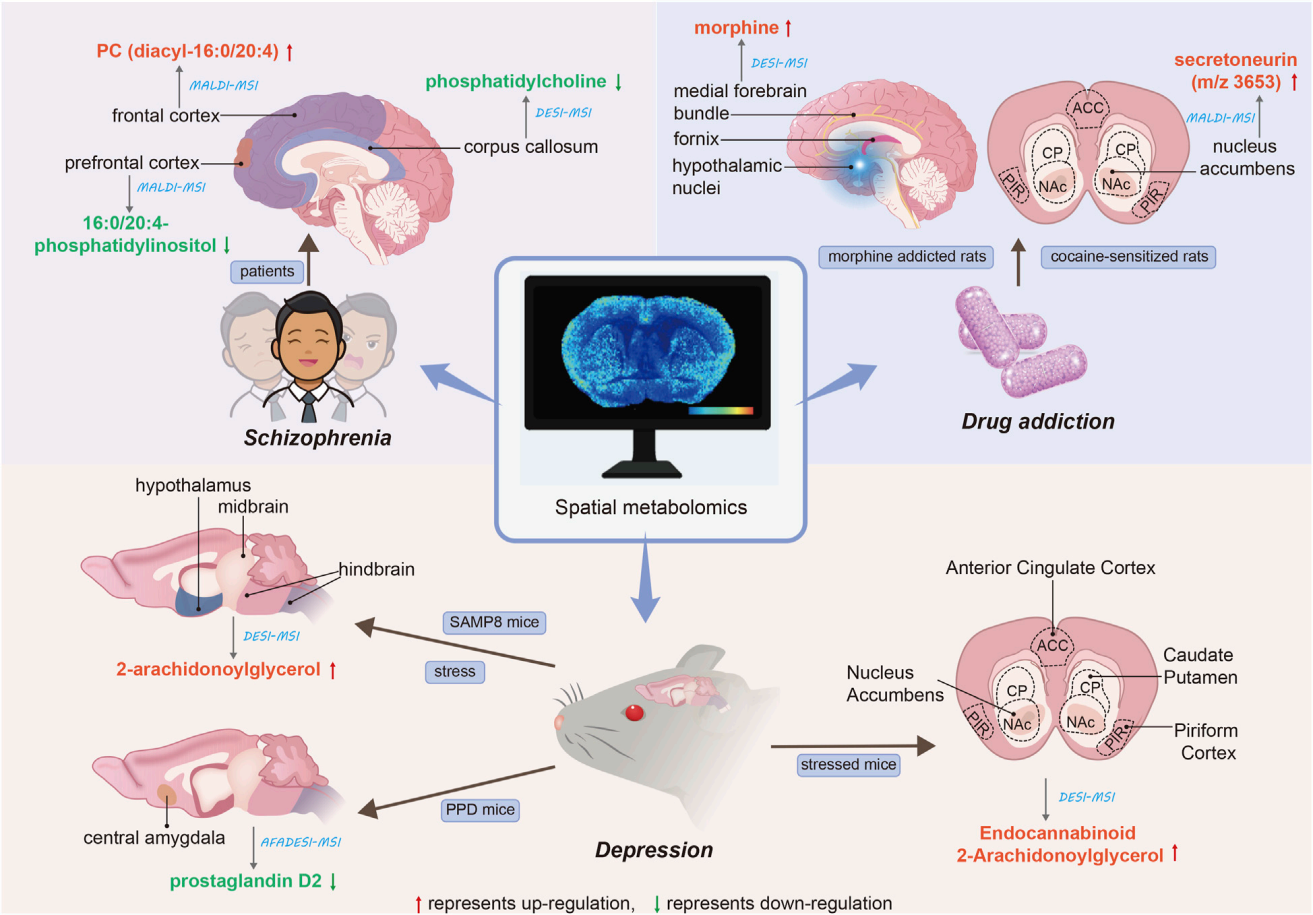
Applications of spatial metabolomics to mental disorders. The red arrows denote the upregulation of the corresponding metabolite, while the green arrows indicate the downregulation of the corresponding metabolite. The straight lines illustrate the spatial distribution of metabolites within brain tissue. The blue italics represent the analytical techniques employed, and the blue text boxes indicate the research subjects. The black italics denote the type of mental disorder.

Drug addiction remains a significant global health concern. Researchers (Uys et al., 2010) employed a combination of MALDI-MSI tissue mapping, MALDI-MSI tissue imaging, and bioinformatics analysis to discern differences in protein expression and localization in the nucleus accumbens (NAc) of cocaine-sensitized rats. Through additional sequencing experiments via MALDI tandem mass spectrometry and a database search of measurement quality, they identified an increase in expression of secretoneurin (m/z 3653). Moreover, the distribution of secretoneurin in the NAc was determined through MALDI tissue imaging, and the heightened expression of its precursor protein, secreted granuloprotein II, was verified via Western blotting. This spatial localization aligns with previous immunolocalization studies of secreted neurotin (Marksteiner et al., 1993). Prolonged exposure to morphine can lead to the development of addictive behaviors, and early diagnosis may mitigate the adverse effects of these behaviors on individuals and society. The authors (Bodzon-Kulakowska et al., 2016) utilized the brains of morphine-addicted rats for DESI analysis. Following morphine administration, the substance exhibited marked overexpression in the medial forebrain bundle, hypothalamic nuclei, and fornix region. Furthermore, two systems (BioMap, Datacube) were utilized to analyze images of rat brain tissue under morphine and compare their ease of use and the quality of results obtained. The ST (22:0) ratio of morphine to control rat brain peak intensity was 3.44 for BioMap and 3.55 for Datacube. Although the results were similar, the authors posit that BioMap proves more beneficial for DESI IMS analysis. The application of spatial metabolomics to mental disorders is summarized in Figure 1.

## 5 Spatial metabolomics, Chinese medicine and mental disorders

Chinese medicine has unique advantages in psychiatric disorders and the adverse effects of antipsychotic drugs. Therefore, Chinese medicine’s efficacy in treating psychiatric disorders has been gradually emphasized in clinical practice. The application of antipsychotic drugs is still the primary treatment for mental disorders at this stage. However, poor patient compliance, a sense of discrimination, adverse drug reactions, and complex interactions between different drugs often adversely affect clinical efficacy during treatment. Moreover, almost all the essential principles of drug action established in Western psychopharmacology in the 20th century were discovered empirically in TCM during the 2000 years of evolution (Shorter and Segesser, 2013). In recent years, researchers have made significant progress in basic research and clinical treatment of mental disorders based on TCM characteristics. In today’s clinical therapeutic practice, TCM therapy combined with Western medicine is mainly used for treatment, which TCM treatment is diverse, including but not limited to decoction, Chinese patent drug, acupuncture, TCM gongfu (Baduanjin, Qigong, and Tai-Chi) and Five-Element Music (Xu et al., 2011; Lin et al., 2012; Chan et al., 2015; Zhao et al., 2019; Li et al., 2022; Lam et al., 2024; Zhou, 2020; Wu et al., 2024; Chen et al., 2015; Yeung A. et al., 2018; Zou et al., 2018).

TCM has a complex and diffuse composition, and its formulation is a complex combination of several natural medicines. The study of TCM on the etiology and pathogenesis of mental disorders is still at an exploratory stage. In recent years, accumulated studies have revealed the application of spatial metabolomics approaches to study the etiology and pathophysiology of complex systemic disorders, including depression and other psychiatric disorders, as well as the mechanisms of TCM effects. However, a single “metabonomics” technique may not fully reflect the mechanisms by which TCM treats mental disorders. Therefore, in the study of mental disorders, data from spatial metabolomics, spatial proteomics, and spatial transcriptomics should be integrated to decipher the biological significance and spatial correlation from differential metabolites, proteins, and genes further to explore the mechanism of TCM for mental disorders. So far, most of the studies on TCM for mental disorders first started with untargeted metabolomics. Then, a series of different endogenous metabolites were obtained from standard controls to infer disease-related metabolic pathways, which provided clues for further mechanistic studies but, at the same time, lacked specificity (Gu et al., 2021). Based on this phenomenon, we propose that future studies should not be limited to full-spectrum metabolites but should also focus on targeted metabolomics for further validation. In addition, each metabolomics platform has its advantages and limitations, and multiple platforms should be clustered to apply for targeted metabolomics studies to obtain different spatial metabolomics data to discover and characterize common biomarkers when conditions allow, which in turn will collectively provide new ideas for the development of antidepressant natural products for psychiatric disorders. Despite the rapid growth in the application of metabolomics for treating psychiatric disorders under TCM interventions (Liu, 2020; Zhu et al., 2024; Lv et al., 2022; Zhou et al., 2020), the application of spatial metabolomics is still in the preliminary research stage. We can foresee that shortly, researchers will vigorously carry out corresponding animal models and even clinical studies in spatial metabolomics research of TCM for the treatment of mental disorders. Meanwhile, under the extensive guidance of spatial metabolomics, TCM is expected to become a more acceptable therapeutic option for treating mental disorders.

## 6 Challenges and perspectives

Today, the unique position of spatial metabolomics in the field of nervous system research is widely acknowledged, and it has begun to find application in studying the metabolic mechanisms of human mental diseases and in the development of new drugs. Serving as a breakthrough technology, spatial metabolomics has opened up numerous new opportunities for the molecular diagnosis of mental diseases treated with traditional Chinese medicine. Nevertheless, it also encounters various challenges, such as metabolite identification and chromatographic separation, as well as issues related to mass spectrometry databases and data sharing (Collins et al., 2021).

Fortunately, advancements in instrumentation, experimental techniques, and analytical software have helped alleviate many of these challenges. For instance, researchers can overlay MS images with optical or HE scans and focus on tissue microregions or lesions of interest to accurately extract mass spectrometry data for the target region in metabolic studies. This approach mitigates the challenges associated with the difficult isolation of study specimens (Jiang H. et al., 2022). In future studies, we can explore three-dimensional MSI, construct multiple slices of two-dimensional MSI data, and visualize another dimension of drug distribution. Furthermore, the biological computing challenges associated with increased spatial resolution also necessitate the development of more efficient data mining tools (Angel and Caprioli, 2013).

It can be predicted that multi-omics joint analysis will become a key research strategy in the future. This approach not only mitigates the data deficiencies stemming from data noise and missingness in single omics analysis, but also reduces the false positive outcomes generated by single omics analysis through the mutual verification of multiple omics data resources. Consequently, multi-omics joint analysis is more conducive to systematically analyzing the multifaceted mechanisms or phenotypic connections of biological models at various levels and perspectives. Moreover, it facilitates the collaborative exploration of potential regulatory network mechanisms within organisms (Zheng et al., 2023).

Specifically, in-depth studies on the spatial distribution of active/toxic ingredients and their metabolites about different metabolites *in vivo* will be carried out to clarify the active/poisonous ingredients and their target areas and to elucidate the mechanisms of the efficacy or toxicity of traditional Chinese medicines more accurately. We can combine spatial metabolomics with spatial proteomics and spatial transcriptomics to realize multi-dimensional studies on quality control, metabolic distribution, and pharmacodynamic or toxicity mechanisms of TCM at metabolic, protein, and gene levels. The blood-brain barrier maintains the relative stability of the intracerebral environment and blocks drug molecules outside the barrier. The combination of MSI and 3D imaging is also strategically important in studying the intracerebral distribution of drugs and neurological side effects.

Currently, spatial metabolomics has shown vigorous development in exploring the metabolic mechanisms of the nervous system. However, the nascent application of traditional Chinese medicine in the treatment of mental diseases remains underdeveloped. It is worthwhile to expect that MSI technology will provide a new vision for treating mental disorders in Chinese medicine, and the application of spatial metabolomics in treating mental disorders in Chinese medicine will become a key research direction.

Therefore, further research is imperative, as it holds significant guiding implications for studying the metabolic mechanisms underlying TCM treatment of mental diseases. In summary, there exists substantial room for the development of spatial metabolomics in the realm of traditional Chinese medicine and mental illness. Through continual refinement and innovation, it can significantly contribute to the modernization of traditional Chinese medicine.
